# Manual of Skin Diseases

**Published:** 1892-09

**Authors:** 


MANUAL OF SKIN DISEASES.—With special reference to diagnosis and treatment, for the use of students and general practitioners. By W. A. Hardaway, M. D , Professor of Skin Diseases, in the Missouri Medical College and in the St. Louis Post-Graduate School of Medicine; Dermatologist to the Augusta Free Hospital for Children; Consulting Dermatologist to the City, and Female Hospitals, etc. Published by Lea Brothers & Co., Philadelphia, Pa.

PROFESSOR W. A. HARDAWAY has written a very condensed, instructive, and admirably arranged treatise on the Diseases of the Skin. His style and descriptions are good, and though in some instances he might elaborate, yet for the rushing student and busy practitioner much knowledge can be obtained. His appendices are quite in good taste and handy as references, for many of us are at times in need of a good suggestion as to a formula, when ours have failed to do the work, and here good ones can be found with the minimum exertion. We take pleasure in recommending this volume.

